# Quality of Life Prior and in the Course of the COVID-19 Pandemic: A Nationwide Cross-Sectional Study with Brazilian Dietitians

**DOI:** 10.3390/ijerph18052712

**Published:** 2021-03-08

**Authors:** Raquel Adjafre da Costa Matos, Rita de Cassia Coelho de Almeida Akutsu, Renata Puppin Zandonadi, Raquel Braz Assunção Botelho

**Affiliations:** Department of Nutrition, Faculty of Health Sciences, University of Brasilia, Brasilia 70910-900, Brazil; raquel.adjafre@gmail.com (R.A.d.C.M.); rita.akutsu@gmail.com (R.d.C.C.d.A.A.); renatapz@unb.br (R.P.Z.)

**Keywords:** dietitian, quality of life, SARS-COV-2, pandemic, health

## Abstract

Dietitians as healthcare professionals could decrease their quality of life during the SARS-COV-2 pandemic period; therefore, this study aimed to compare Brazilian dietitians’ perceptions of quality of life before and during the pandemic. This nationwide cross-sectional research aimed to evaluate Brazilian dietitians’ quality of life before and in the course of the COVID-19 pandemic, using a previously validated self-administered instrument WHO-QOL-BREF in Brazilian-Portuguese. The questionnaire was composed of 26 items (four domains) to evaluate life quality (physical, psychological, social relationship, and environment). The questionnaire also presented some sociodemographic variables and three questions about the COVID-19 pandemic. It was applied using GoogleForms^®^ platform (Google LLC, Mountain View, CA, USA). For the statistical analysis of data, Paired T-test, Chi-squared test, and Analysis of Variance were used. A total of 1290 Brazilian dietitians replied to the instrument. Comparing quality of life (QoL) before SARS-COV-2 (3.83 ± 0.59) and during the pandemic (3.36 ± 0.66), data was statistically different. Comparing prior and in the course of the COVID-19 pandemic, all variables and domains presented statistical differences (better before the pandemic period). Among Brazilian dietitians, the psychological health domain was the most affected. The Sars-Cov-2 pandemic negatively impacted the QoL of Brazilian dietitians since health professionals face changes in their lives because of work.

## 1. Introduction

There are many challenges involving the healthcare working environment and personal life [1], mainly in the unexpected COVID-19 pandemic [2,3,4]. The pandemic led governments to take severe mitigation measures, including community-wide lockdowns, home quarantines, home-working, social distancing, and the barring of social meetings to minimize the spread of the virus [5]. The COVID-19 is a new disease with a high transmission rate, presenting a greater risk of infection for healthcare professionals [6]. Estimates suggest that healthcare workers could account for 20% of all diagnoses [7], bringing the fear and risk of death from the viral infection and unbearable psychological pressure [4,8], which can affect their quality of life [9]. Healthcare professionals face the quotidian task of providing thoughtful care to patients experiencing a range of health burden conditions, often on the background of complex medical, social, and psychological issues added by difficulties posed by increasingly limited healthcare resources required to provide a high-quality and evidence-based service, representing a challenge to them [1]. A study performed in India showed a high prevalence of symptoms of depression and low quality of life perception among health care professionals during the pandemic [9]. This study showed that stressors related to the health professionals’ work environment during the pandemic could be a key-driver of depression and anxiety [9]. Additionally, depression and anxiety during the pandemic were associated with low quality of life (QoL) [9].As healthcare professionals, dietitians are probably facing difficulties in their work and life during the pandemic, as the fear of Sars-CoV-2, low wages, unemployment, lack of recognition, barrier in geographical mobility and distancing [10,11,12]. The uncertainty about the Sars-CoV-2, itself and their job insecurity may affect the dietitians’ QoL. There are studies on patients’ QoL during the Sars-CoV-2 pandemic [13,14,15,16,17], but few among health professionals’ QoL during the pandemic [18]. Dietitians apply scientific knowledge about food and nutrition to promote optimal health outcomes to individuals, groups, and communities in health and disease states [19,20]. They are the qualified professionals able to prescribe and manage therapeutic diets for individuals, making them the ideal health professional workforce to address nutrition risk factors and stimulate the patient’s recovery based on nutrient intake [19,20].

In Brazil, dietitians present a vast assortment of work options, from working in hospitals with inpatients or leading hospital restaurants or working in clinics, schools, and food services [21]. These environments can bring direct contact with infected people, presenting challenges in this new work scenario. Several food services closed, leading to unemployment and facing many reopening risks when permitted. As health professionals, the dietitians work directly with people infected with the Sars-CoV-2 during de pandemic. Frequently, their risks are belittled by other health professionals and the Government, impacting their QoL. Recent Brazilian Law [22] dealing with the protection of professionals exposed to the risks of Sars-Cov-2 did not include dietitians on its lists.

Considering that, we hypothesized that dietitians’ QoL would decrease during the pandemic period. Several studies were conducted on health professionals’ QoL during the pandemic [9,18,23,24], but ours is the first on dietitians’ QoL. Additionally, there are few studies on dietitians’ QoL [1,25] before the pandemic. Therefore, we aimed to compare Brazilian dietitians’ QoL prior and in the course of the COVID-19 pandemic to identify the factors that influence dietitians’ QoL in these moments, helping them retrieve after this period.

## 2. Materials and Methods

### 2.1. Study Design and Instrument

This nationwide cross-sectional study was carried out by applying a previously validated self-administered questionnaire WHO-QOL-BREF [14] in Brazilian-Portuguese [15] to investigate the Brazilian dietitians’ QoL prior to and in the course of the pandemic. The instrument was composed of 26 items to evaluate the QoL in 4 domains (physical—7 items, psychological—6 items, social relationship—3 items, and the environment—8 items). Researchers included questions to gather socioeconomic and demographic characteristics and three questions related to the pandemic (do you continue working during the Sars-CoV-2 pandemic?, did you test positive for Sars-CoV-2?, and did anybody in your family test positive for Sars-CoV-2?). The complete instrument was applied using GooleForms^®^ access link sent by email, apps, and social networks. Participants were invited to participate from 26 May–7 June 2020. Volunteers from all Brazilian regions were recruited with the help of the Dietitians Councils, support groups, and media outreach to reach as many dietitians as possible. Volunteers received the research link, the invitation to participate, and the consent form.

### 2.2. Participants and Ethics

Researchers invited dietitians from the five Brazilian regions to participate and to trace their QoL before and during the Sars-CoV-2. The Ethics Committee of the University of Brasília approved the study (protocol No.54822316.1.00000030). The study followed the Declaration of Helsinki guidelines and the Recommendations of Scholarly work in Medical Journals. Data from the Brazilian Federal Dietitians Council was used to calculate the sample size. At the time of the study, the Council presents 129,134 registered dietitians [26], and the minimum representative sample size would be 1059 dietitians [26], considering an error (e) of 3% and a level of significance (α) of 5% [27]. The inclusion criteria were to be a dietitian living and working in Brazil.

### 2.3. Statistical Analysis

IBM SPSS Statistics for Windows (Armonk, NY: IBM Corp) were used to analyze data on measures of central tendency and sample dispersion; comparisons of samples’ means and proportions through paired T-test, Chi-squared test, and Analysis of Variance (ANOVA); and Cronbach’s alpha to evaluate the instrument reliability. The Shapiro-Wilk test was used to test the normality of the continuous variables.

## 3. Results

Table 1 presents the participants’ characteristics. A representative sample of 1290 dietitians from the five Brazilian regions participated in the study. They were mostly female (92.5%), Catholic (53.1%), aged from 25 to 39 years old (58.8%), with a partner (64.2%), and without children (58.3%). Most of them continued working during the pandemic (84.6%) and were not diagnosed with COVID-19 (96%), nor did their relatives (80.2%). Data from the dietitians’ QoL prior and in the course of the pandemic period compared by their characteristics are presented in Table 2. In general, and for all variables, QoL before the pandemic was 3.83 ± 0.59, statistically different (*p* = 0.000) from the period in the course of the pandemic (3.36 ± 0.66). A comparison between prior and in the pandemic course presented statistical differences for all variables (lower during the pandemic) (*p* < 0.05). The normality test showed that the samples are normal for the continuous variables.

Before the pandemic, some variables (gender; having children; area of practice; the number of workplaces; and type of institution where the dietitians finished their undergraduate degree) did not influence their QoL (Table 2). It was similar to the period during the pandemic, except for the area of practice. Dietitians working in the teaching area presented a better QoL than the others (*p* = 0.000). Before the pandemic period, teaching did not differ from other areas of practice. Dietitians from the South and Southeast regions presented the best scores to QoL during the pandemic period, and the North and Northeast regions presented the worst scores to QoL. Those from the North region presented worse QoL than Midwest (*p* = 0.028), South (*p* = 0.001), and Southeast (*p* = 0.001), similar to before the pandemic. The South region dietitians’ QoL was better than the Midwest (*p* = 0.022), Northeast (*p* = 0.002), and North (*p* = 0.000) regions (Table 2).

The dietitians with family income >5 minimum wages presented better QoL than those with lower family income during the pandemic period (*p* = 0.000) (Table 2). Before the pandemic period, the dietitians with a family income >10 minimum wages presented better QoL than those with family income up to 10 minimum wages (*p* = 0.000). In both periods, Ph.D. dietitians have a higher QoL than the others (*p* =0.000). However, before the pandemic, Master dietitians differed from Graduates (*p* = 0.001) but did not differ from those with specialization (*p* = 0.085). During the pandemic period, only the Ph.D. dietitians differed from the others on QoL (*p* = 0.000 for graduates and specialization; *p* = 0.003 for Master). Dietitians with more than 15 years from undergraduate completion presented better QoL than those up to 5 years before the pandemic (*p* = 0.001). During the pandemic period, >10 years from the undergraduate completion presented better QoL than the ones up to 2 years of undergraduate completion (*p* = 0.006). When stratifying data relating to the time of undergraduate completion, family income, and the work area, dietitians with more than ten years of graduation, more than 20 minimum wages, and in the teaching area presented the highest QoL scores (4.64 ± 0.11 before the pandemic and 4.12 ± 0.12 during the pandemic). People who test positive or not for Sars-CoV-2 did not differ the QoL and people whose relatives test positive or not for Sars-CoV-2. Dietitians who changed their work type because of Sars-CoV-2 presented better QoL than those who are not working or are working in person.

Evaluating the QoL by domains (Table 3), all of them presented lower means during the pandemic than the period prior to the COVID-19 pandemic (*p* < 0.05). The second domain (psychological health) is the most affected among Brazilian dietitians before and in the course of the pandemic (*p* < 0.05). Before the COVID-19 pandemic, the means of domain 1 (physical health) was higher than the others (*p* < 0.05), but during the pandemic period, it did not differ from domains 3 and 4 (Table 3). The Cronbach alpha of the general instrument was 0.925, and for domain 1, it was 0.719; domain 2, 0.798; domain 3, 0.802; and domain 4, 0.773, showing good reliability for the entire instrument and each domain.

Before the pandemic period, the best means were for questions 13 (How available to you is the information that you need in your day-to-day life?), 6 (To what extent do you feel your life to be meaningful?), and 15 (How well are you able to get around?), and during the pandemic, the same questions were the ones with highest scores but in a different order: questions 6, 13, and 15. The worse means were for questions 12 (Have you enough money to meet your needs?), 21 (How satisfied are you with your sex life?), and 5 (How much do you enjoy life?) before the pandemic. During the pandemic period, the worse means were for questions 4, 14, and 5 (How much do you need any medical treatment to function in your daily life?; To what extent do you have the opportunity for leisure activities?; and How much do you enjoy life?). Only question number 5 remains, presenting one of the worst means.

Comparing the domains of quality of life before the pandemic, domain 1 differed from all other domains (0 = 0.000 comparing to domains 2 and 3; *p* = 0.040 comparing domain 4). After the pandemic, domain 1 only differed from domain 2 (*p* = 0.000). Mean QoL for domain 1 was equal to domain 3 (*p* = 0.099) and domain 4 (*p* = 0.202).

## 4. Discussion

Our hypothesis that the COVID-19 pandemic would negatively affect Brazilian dietitians’ quality of life was confirmed. When the pandemic of COVID-19 reached Brazil, the country was at a time of economic stagnation, problems with health and social protection systems, difficulties in food security programs, an accelerated increase in poverty, and, above all, extreme poverty and a significant increase in the vulnerable population. Since March 2020, Brazil has accumulated a drop in the Gross Domestic Product (GDP) [28]. This decline, partly caused by social isolation during the pandemic, has significantly increased formal and informal unemployment and labor relations’ precariousness. This new scenario directly impacts the dietitian’s work and quality of life, not only on his working conditions, income, and uncertainties but also on the feeling of powerlessness in the country’s face of hunger.

Dietitian acts at all levels of complexity in the health system and can potentially reduce the risk of worsening the disease and contribute to the recovery of patients affected by COVID-19. This study is the first on dietitians’ QoL comparing prior and in the course of the COVID-19 pandemic period. In general, QoL before Sars-Cov-2 was higher than the pandemic period (*p* < 0.05) (Table 2). Of a total of 1290 Brazilian dietitians, most of the participants were female (92.5%). The high percentage of females among dietitians is typical [21,29,30,31], but gender did not influence QoL in our study. Table 2 showed that, before and during the pandemic period, Ph.D. dietitians have a higher perception of QoL than the others (*p* < 0.05). During the pandemic period, only the Ph.D. dietitians differed from the others on QoL perception. Most of the Ph.D. dietitians work in the teaching area. During the pandemic period, individuals who work teaching presented better QoL than other practice areas (clinic, foodservice administration, public health, and others). Dietitians with family income > 20 MW, working in the teaching area for more than ten years, presented the best Qol perception before and during the pandemic, but this is a small subsample of the studied dietitians (*n* = 67).

In general, and for all variables, QoL before SARS-COV-2 was better than during the pandemic period (*p* < 0.05). It was expected since Sars-Cov-2 brought the dread and hazard of death, psychological pressure, fear of losing family members, social isolation, unemployment, and several other unexpected changes in life [4,8]. Dietitians with partners have higher QoL means before and during the pandemic than dietitians without partners, probably because matrimony is associated with higher life happiness and welfare, related to better health and life expectancy [32]. Marriage is associated with well-being, QoL [32,33], and highest financial status and education [34]. QoL results did not differ before or during the pandemic when evaluating dietitians with and without children. Having a partner brought a better QoL, not influenced by having children. Mo et al. [35] discuss that health professionals need to have more time away from their loved ones during the pandemic, and this probably decreased QoL, especially for domain 2 (psychological aspects).

About 15% of the dietitians did not continue working during the pandemic (Table 1). These professionals followed the tendency of the Brazilian official data for general workers (in the same period) in which unemployment increased in Brazil during the pandemic [36]. In the Northeast (from 13.6% in 2019 to 15.6% in 2020), followed by the North region (from 10.6% to 11.4%) and the Southeast (from 11.4% to 12.4%). The increase in unemployment affects family income. Our data confirmed that family income affects QoL’s perception, and dietitians with the lowest ranges of family income had the worse means to QoL (general and domains) (Table 2 and Table 3).

Dietitians that changed their type of work (continuing work in person with some adaptations or remotely) presented better QoL perception than those that are not working or working in person (Table 2). Despite a meta-analytic study [37] suggesting that employed people present better life satisfaction, in the course of the pandemic, people working in-person (dealing with the fear and the risk of COVID-19) are more afraid of becoming infected or transmitting the SARS-CoV-2 to a relative [18], worsening QoL’s perception. Additionally, people facing unemployment tend to suffer from the psychological and economic burden, potentially affecting QoL [4,8]. South and Southeast dietitians presented the best scores to QoL during the pandemic, and the North and Northeast regions presented the worst scores to QoL (Table 1). Among other factors, the North and Northeast regions presented the most significant proportional increase in official cases of SARS-COV-2 in Brazil in the period of our study [38].

The dietitians with family income < 5 mean wages presented worse QoL. Income inequality is probably one of many determinants of QoL perception [39]. In the course of the pandemic, many individuals and their relatives are isolated at home, growing their household expenses (e.g., water, energy, food, and other bills). Higher education levels are also associated with welfare and satisfaction and affect income [40], confirming our results (better QoL scores in dietitians with more education and income).

Our data showed that dietitians who tested positive or not for Sars-CoV-2 did not differ in the QoL and people whose family members tested positive or not for Sars-CoV-2. The disease’s uncertainties make people continue afraid of the Sars-Cov-2 [17,18] despite the first contamination or for their family members. The second domain (psychological health) was the most affected among Brazilian dietitians during the pandemic (*p* < 0.05), confirming that high-stress situations are followed by psychological responses [18]. Before the Sars-Cov-2 pandemic, the QoL means of domain 1 (physical health) was higher than the others (*p* < 0.05), but during the pandemic period, it did not differ from domains 3 and 4 (Table 3). In Brazil, until the moment of the study´s data collection, all the gyms and parks were closed, impairing the practice of physical activity. Additionally, the first domain is related to the work capacity (influenced by the pandemic, as discussed before). Restaurants, stores, and shopping malls were also closed, affecting mobility during the pandemic period. The perception of fatigue and low-energy can be associated with the lack of physical activity and the stress during the pandemic. Considering the items, the worse means were for questions 12 (Have you enough money to meet your needs?), 21 (How satisfied are you with your sex life?), and 5 (How much do you enjoy life?) before the pandemic period. Before and during the pandemic, question 5 (“How much do you enjoy life?”) presented the lowest means, but this was lower during the pandemic period. Since many cities in Brazil closed restaurants, bars, night clubs, and prohibited events, the population did not present many leisure options that influence life enjoyment. Gyms, parks, and shopping malls were also closed. That is why during the pandemic period, question 14 (To what extent do you have the opportunity for leisure activities?) was included as one of the worse means. Questions 4 (How much do you need any medical treatment to function in your daily life?), and 5 also presented lower means during the pandemic. The changes with low means to the responses for question 4 can be related to the use of medications to psychological burden and the medications and supplements (vitamins, minerals, bioactive compounds, amino acids) that supposedly improve body immunity and prevent critical evolution of Sars-Cov-2.

The pandemic in Brazil is also associated with a difficult economic period that can affect the perception of dietitians’ QoL and other population portions. Unsure about the after-days on work conditions, income, and social protection, lowered QoL’s mean scores, mainly the psychological domain. Dietitians are health professionals that faced a complete change in their work environment and are affected by the increase in hunger among the Brazilian population that they try to protect daily as part of their activities. This scenario may have affected their QoL perception. Simultaneously, the pandemic can bring the search for new strategies for health professionals and better conditions in hospitals and clinics. New routines and behaviors for food production can be developed, thinking about food safety within the production area and consumers’ attitudes. The dietitians should show the importance of his/her work to avoid contamination in food services and discuss eating habits and immunity.

Regardless of the period, it is important to emphasize that to improve the quality of life of the dietitians, it is necessary to improve compensation and recognition of the profession, as well as the conditions to work. It is also necessary to strengthen this class’s entities, especially concerning wage levels and labor relations. Health policymakers should discuss health professionals’ role as a multidisciplinary team, highlighting the importance of dietitians for the health system since they still feel little recognized for their work [21]. This study may open the door to further research and discussion in health professionals’ QoL and a clear understanding of the factors that influenced them before and during the pandemic, helping these professionals recover and strengthen after this period.

This study’s potential limitation is the unique tool used to evaluate the studied population’s QoL that does not emphasize depression and anxiety, phenomena that are mainly presenting during the pandemic. However, a previous study showed that depression and anxiety during the pandemic were associated with low quality of life (QoL) [9]. It is also important to highlight that the WHOQOL-BREF can be used as an instrument to support decision-making. It is possible to identify what is efficient and what can be proposed in terms of public policies to improve the quality of life for the dietitians and for the population. Further studies are necessary to verify this association among Brazilian dietitians.

## 5. Conclusions

This study confirmed the hypothesis that the COVID-19 pandemic would negatively affect Brazilian dietitians’ quality of life. The research sample had national representativeness and was similar to the dietitians’ profile described by the Federal Council of Dietitians, with most females, Catholics, between 25 and 39 years old, with a partner without children. Dietitian is the health professional at the forefront of nutritional care for the population. They act at all levels of complexity in the health system and can potentially reduce the risk of worsening the disease and contribute to the recovery of patients affected by SARS-CoV-2. The different spheres (population, governments, and other health professionals) must recognize these professionals’ relevance to the country’s public health. Dietitians and other health professionals had their lives changed in different areas. Our study revealed a significant QoL burden of dietitians working in-person or not working. Before the pandemic, the physical domain presented the best QoL scores and the worst results on the psychological domain.

During the pandemic, the psychological domain continued to be the worst aspect of QoL, and the other domains were impacted equally. Dietitians with more than ten years of graduation completion, working in the teaching area, and higher incomes have higher QoL perception. Gender, children, number of workplaces, and having SARS-COV2 did not influence QoL perception among dietitians. Regions of the country influenced QoL, and this can be related to different wages and work conditions. Better care should be provided to dietitians as health professionals during future life-threatening disease outbreaks, mainly regarding QoL’s psychological aspects. Health policymakers should discuss health professionals’ role as a multidisciplinary team, highlighting each category’s importance for the health system, as dietitians still feel little recognized for their work.

It is important to highlight that at the beginning of this research, we believed that the pandemic would be earlier controlled. However, until now, we are still experiencing the pandemic, which probably has a more substantial impact on the studied constructs. Future studies are necessary to identify dietitians who worked directly with patients with COVID-19 and even if there was a reduction in their salaries.

## Figures and Tables

**Table 1:** Characterization of Brazilian dietitians and SARS-COV-2 questions (*n* = 1290).

VARIABLE		*n*	%
Gender	Female	1192	92.5
	Male	97	7.5
Age group	21 to 24 y/o	135	10.5
	25 to 29 y/o	255	19.8
	30 to 34 y/o	264	20.5
	35 to 39 y/o	238	18.5
	40 to 44 y/o	128	9.9
	45 to 49 y/o	82	6.4
	50 to older	187	14.5
Religion	Catholic	684	53.1
	Protestant	266	20.6
	Spiritism	202	15.7
	Agnostic	69	5.4
	Others	68	5.3
Level of education (highest degree)	Graduate	280	21.7
	Especialization/Residency	640	49.7
	Master	228	17.7
	PhD	141	10.9
Marital status	Without partner	461	35.8
	With partner	828	64.2
Children	Yes	538	41.7
	No	751	58.3
Family monthly income	≤ 1 MW	35	2.7
	> 1 to 2 MW	102	7.9
	>2 to 3 MW	182	14.1
	>3 to 5 MW	284	22.0
	>5 to 10 MW	399	31
	>10 to 20 MW	220	17.1
	>20 MW	67	5.2
Number of household members	1	131	10.5
	2	349	27.9
	3	351	28.1
	4	309	24.7
	>5	109	8.7
Area of Practice	Clinic	310	24
	Foodservice administration	164	12.7
	Public health	111	8.6
	Teaching	99	7.7
	Others	62	4.8
	More than one area of practice	543	42.1
Number of workplaces	1	815	63.2
	2	333	25.8
	3	83	6.4
	>3	58	4.5
Type of institution where you finished your undergraduate degree	Public	625	48.5
	Private	664	51.5
Time from the undergraduate completion	≤2 years	265	20.6
	>2 to 5 years	221	17.1
	>5 to 10 years	243	18.9
	>10 to 15 years	243	18.9
	>15 years	317	24.6
Do you continue working during Sars-CoV-2?	No	198	15.4
	yes remotely	468	36.3
	yes, in person with some adaptations	344	26.7
	yes in person	279	21.6
Did you test positive for Sars-CoV-2?	No	1237	96.0
	Yes	52	4.0
Did any family members test positive for Sars-CoV-2?	No	1034	80.2
	Yes (does not live with me)	67	5.2
	Yes (living with me)	188	14.6

MW—Minimum Wage in Brazil (7 June 2020)—U$213.0.

**Table 2:** Brazilian dietitians Quality of life by socioeconomic and demographic variables prior and in the course of the pandemic period (*n* = 1290).

VARIABLE		Before * Pandemic	During * Pandemic
		Mean ± SD	Mean ± SD
Gender	Female	3.82 ^a^ ± 0.59	3.35 ^a^ ± 0.66
	Male	3.89 ^a^ ± 0.61	3.44 ^a^ ± 0.69
Age group	21 to 24 y/o	3.76 ^ab^ ± 0.61	3.25 ^ab^ ± 0.63
	25 to 29 y/o	3.74 ^a^ ± 0.60	3.22 ^a^ ± 0.67
	30 to 34 y/o	3.83 ^ab^ ± 0.64	3.35 ^abc^ ± 0.68
	35 to 39 y/o	3.83 ^ab^ ± 0.58	3.40 ^bc^ ± 0.65
	40 to 44 y/o	3.85 ^ab^ ± 0.63	3.36 ^abc^ ± 0.73
	45 to 49 y/o	3.90 ^ab^ ± 0.43	3.43 ^abc^ ± 0.54
	50 to older	3.93 ^b^ ± 0.53	3.53 ^bc^ ± 0.60
Brazilian region	North	3.71 ^a^ ± 0.61	3.19 ^a^ ± 0.67
	Northeast	3.82 ^abc^ ± 0.62	3.31 ^abc^ ± 0.66
	Midwest	3.81 ^ab^ ± 0.58	3.36 ^bc^ ± 0.66
	Southeast	3.88 ^b^ ± 0.57	3.43 ^bcd^ ± 0.64
	South	3.96 ^bc^ ± 0.53	3.55 ^d^ ± 0.60
Religion	Catholic	3.85 ^ac^ ± 0.60	3.38 ^ab^ ± 0.65
	Protestant	3.71 ^bc^ ± 0.59	3.26 ^a^ ± 0.64
	Spiritism	3.89 ^ac^ ± 0.53	3.40 ^ab^ ± 0.64
	Agnostic	3.79 ^abc^ ± 0.60	3.27 ^ab^ ± 0.70
	Others	3.90 ^abc^ ± 0.61	3.50 ^b^ ± 0.74
Marital status	Without partner	3.74 ^a^ ± 0.61	3.23 ^a^ ± 0.67
	With partner	3.88 ^b^ ± 0.57	3.42 ^b^ ± 0.64
Children	Yes	3.84 ^a^ ± 0.58	3.39 ^a^ ± 0.64
	No	3.81 ^a^ ± 0.60	3.33 ^a^ ± 0.67
Family monthly income	≤1 MW	3.50 ^a^ ± 0.66	3.15 ^a^ ± 0.71
	>1 to 2 MW	3.64 ^a^ ± 0.66	3.08 ^a^ ± 0.70
	>2 to 3 MW	3.66 ^a^ ± 0.64	3.18 ^a^ ± 0.69
	>3 to 5 MW	3.72 ^a^ ± 0.57	3.24 ^a^ ± 0.64
	>5 to 10 MW	3.90 ^b^ ± 0.55	3.44 ^b^ ± 0.63
	>10 to 20 MW	4.05 ^c^ ± 0.50	3.58 ^b^ ± 0.58
	>20 MW	4.00 ^c^ ± 0.52	3.60 ^b^ ± 0.60
Level of education (highest degree)	Graduate	3.70 ^a^ ± 0.60	3.25 ^a^ ± 0.67
	Specialization/Residency	3.79 ^ab^ ± 0.59	3.33 ^a^ ± 0.65
	Master´s	3.90 ^b^ ± 0.58	3.37 ^a^ ± 0.67
	PhD	4.13 ^c^ ± 0.46	3.64 ^b^ ± 0.56
Area of Practice	Clinic	3.82 ^a^ ± 0.60	3.35 ^a^ ± 0.64
	Teaching	4.06 ^a^ ± 0.49	3.54 ^b^ ± 0.60
	Foodservice administration	3.79 ^a^ ± 0.58	3.34 ^a^ ± 0.67
	Public health	3.84 ^a^ ± 0.61	3.35 ^a^ ± 0.57
	More than one area of practice	3.80 ^a^ ± 0.60	3.33 ^a^ ± 0.69
	Others	3.81 ^a^ ± 0.61	3.39 ^ab^ ± 0.59
Number of workplaces	1	3.81 ^a^ ± 0.59	3.34 ^a^ ± 0.66
	2	3.85 ^a^ ± 0.58	3.34 ^a^ ± 0.64
	3	3.90 ^a^ ± 0.60	3.45 ^a^ ± 0.66
	>3	3.88 ^a^ ± 0.67	3.52 ^a^ ± 0.71
Type of institution where you finished your undergraduate degree	Public	3.86 ^a^ ± 0.56	3.38 ^a^ ± 0.63
	Private	3.80 ^a^ ± 0.62	3.33 ^a^ ± 0.68
Time from the undergraduate completion	≤2 years	3.73 ^a^ ± 0.61	3.22 ^a^ ± 0.67
	>2 to 5 years	3.77 ^a^ ± 0.63	3.33 ^ab^ ± 0.68
	>5 to 10 years	3.86 ^ab^ ± 0.61	3.32 ^ab^ ± 0.66
	>10 to 15 years	3.85 ^ab^ ± 0.60	3.42 ^b^ ± 0.68
	>15 years	3.91 ^b^ ± 0.51	3.46 ^b^ ± 0.60
Do you continue working during Sars-CoV-2?	No	3.72 ^a^ ± 0.64	3.20 ^a^ ± 0.69
	yes in person	3.73 ^a^ ± 0.55	3.25 ^a^ ± 0.62
	yes, in person with some adaptations	3.86 ^b^ ± 0.61	3.40 ^b^ ± 0.66
	yes remotely	3.90 ^b^ ± 0.57	3.45 ^b^ ± 0.65
Did you test positive for Sars-CoV-2?	No	3.83 ^a^ ± 0.59	3.36 ^a^ ± 0.66
	Yes	3.74 ^a^ ± 0.54	3.21 ^a^ ± 0.60
Did any family members test positive for Sars-CoV-2?	No	3.83 ^a^ ± 0.61	3.37 ^a^ ± 0.67
	Yes (does not live with me)	3.80 ^a^ ± 0.55	3.25 ^a^ ± 0.64
	Yes (living with me)	3.84 ^a^ ± 0.53	3.31 ^a^ ± 0.61

* All results are statistically different comparing the period before and during the pandemic. Different lowercase letters (^a,b,c,d^) inside each column and each variable show statistically different results (*p* < 0.05); y/o—years old; MW—Minimum Wage—U$213.0.

**Table 3:** Quality of life domains by socioeconomic and demographic variables of Brazilian dietitians before and during the pandemic period (*n* = 1290).

VARIABLE		Before Pandemic Mean ± SD				During Pandemic Mean ± SD			
		Domain 1	Domain 2	Domain 3	Domain 4	Domain 1	Domain 2	Domain 3	Domain 4
Gender	Female	3.91 ± 0.63	3.10 ± 0.63	3.74 ± 0.84	3.80 ± 0.70	3.40 ± 0.63	2.57 ± 0.74	3.38 ± 0.95	3.39 ± 0.75
	Male	4.07 ± 0.56	3.22 ± 0.64	3.75 ± 0.88	3.83 ± 0.77	3.61 ± 0.66	2.78 ± 0.77	3.28 ± 0.99	3.47 ± 0.85
Age group	21 to 24 y/o	4.03 ± 0.58	2.98 ± 0.64	3.76 ± 0.95	3.60 ± 0.75	3.47 ± 0.56	2.38 ± 0.76	3.25 ± 0.99	3.25 ± 0.76
	25 to 29 y/o	3.92 ± 0.59	3.02 ± 0.63	3.68 ± 0.89	3.68 ± 0.76	3.36 ± 0.61	2.42 ± 0.70	3.27 ± 1.01	3.23 ± 0.81
	30 to 34 y/o	3.93 ± 0.65	3.09 ± 0.65	3.75 ± 0.85	3.84 ± 0.73	3.39 ± 0.66	2.59 ± 0.74	3.36 ± 0.96	3.43 ± 0.77
	35 to 39 y/o	3.87 ± 0.63	3.17 ± 0.65	3.73 ± 0.84	3.86 ± 0.69	3.41 ± 0.65	2.67 ± 0.75	3.42 ± 0.92	3.47 ± 0.73
	40 to 44 y/o	3.92 ± 0.65	3.11 ± 0.59	3.69 ± 0.84	3.86 ± 0.66	3.42 ± 0.63	2.57 ± 0.79	3.30 ± 1.01	3.45 ± 0.76
	45 to 49 y/o	3.92 ± 0.50	3.25 ± 0.50	3.82 ± 0.71	3.87 ± 0.55	3.48 ± 0.62	2.74 ± 0.65	3.50 ± 0.74	3.39 ± 0.66
	50 to older	3.92 ± 0.68	3.24 ± 0.59	3.83 ± 0.78	3.93 ± 0.63	3.49 ± 0.66	2.79 ± 0.70	3.55 ± 0.89	3.54 ± 0.71
Brazilian region	North	3.83 ± 0.66	2.97 ± 0.64	3.62 ± 0.91	3.67 ± 0.70	3.25 ± 0.62	2.38 ± 0.76	3.20 ± 1.01	3.19 ± 0.75
	Northeast	3.92 ± 0.64	3.11 ± 0.64	3.75 ± 0.86	3.77 ± 0.76	3.38 ± 0.62	2.55 ± 0.73	3.39 ± 0.97	3.33 ± 0.78
	Midwest	3.95 ± 0.60	3.08 ± 0.60	3.73 ± 0.84	3.75 ± 0.70	3.47 ± 0.61	2.60 ± 0.73	3.34 ± 0.94	3.39 ± 0.78
	Southeast	3.93 ± 0.63	3.24 ± 0.63	3.82 ± 0.79	3.90 ± 0.67	3.47 ± 0.64	2.72 ± 0.76	3.48 ± 0.91	3.50 ± 0.73
	South	4.00 ± 0.55	3.17 ± 0.57	3.81 ± 0.79	4.03 ± 0.59	3.56 ± 0.64	2.69 ± 0.68	3.45 ± 0.87	3.66 ± 0.65
Religion	Catholic	3.94 ± 0.64	3.14 ± 0.64	3.76 ± 0.86	3.82 ± 0.71	3.44 ± 0.63	2.59 ± 0.75	3.40 ± 0.95	3.42 ± 0.76
	Protestant	3.88 ± 0.62	3.01 ± 0.62	3.66 ± 0.89	3.60 ± 0.71	3.36 ± 0.62	2.51 ± 0.74	3.32 ± 0.95	3.18 ± 0.73
	Spiritism	3.94 ± 0.56	3.12 ± 0.57	3.75 ± 0.74	3.96 ± 0.60	3.40 ± 0.60	2.58 ± 0.70	3.36 ± 0.91	3.55 ± 0.71
	Agnostic	3.88 ± 0.61	3.08 ± 0.61	3.73 ± 0.83	3.78 ± 0.74	3.35 ± 0.64	2.55 ± 0.72	3.21 ± 1.02	3.36 ± 0.81
	Others	3.95 ± 0.66	3.20 ± 0.62	3.86 ± 0.81	3.92 ± 0.74	3.53 ± 0.73	2.79 ± 0.75	3.50 ± 1.04	3.59 ± 0.87
Number of household members	1	3.88 ± 0.62	3.11 ± 0.66	3.71 ± 0.85	3.80 ± 0.71	3.39 ± 0.68	2.61 ± 0.78	3.31 ± 0.93	3.43 ± 0.75
	2	3.95 ± 0.61	3.10 ± 0.57	3.76 ± 0.79	3.82 ± 0.65	3.46 ± 0.61	2.58 ± 0.75	3.47 ± 0.91	3.40 ± 0.75
	3	3.92 ± 0.61	3.14 ± 0.62	3.81 ± 0.85	3.86 ± 0.72	3.42 ± 0.62	2.63 ± 0.70	3.44 ± 0.98	3.46 ± 0.77
	4	3.92 ± 0.66	3.11 ± 0.65	3.72 ± 0.87	3.75 ± 0.73	3.39 ± 0.64	2.55 ± 0.75	3.29 ± 0.94	3.35 ± 0.76
	>5	3.91 ± 0.63	3.06 ± 0.66	3.60 ± 0.90	3.70 ± 0.75	3.41 ± 0.66	2.51 ± 0.74	3.19 ± 0.98	3.24 ± 0.78
Level of education (highest degree)	Undergraduate	3.89 ± 0.64	3.00 ± 0.63	3.72 ± 0.86	3.56 ± 0.69	3.40 ± 0.66	2.50 ± 0.75	3.33 ± 0.95	3.16 ± 0.78
	Graduate/Residency	3.90 ± 0.63	3.07 ± 0.63	3.67 ± 0.87	3.76 ± 0.71	3.40 ± 0.63	2.56 ± 0.73	3.31 ± 0.95	3.37 ± 0.75
	Master´s	3.95 ± 0.61	3.18 ± 0.62	3.77 ± 0.79	3.97 ± 0.67	3.41 ± 0.63	2.57 ± 0.76	3.38 ± 0.95	3.51 ± 0.74
	PhD	4.07 ± 0.57	3.43 ± 0.48	4.07 ± 0.68	4.20 ± 0.51	3.54 ± 0.59	2.87 ± 0.65	3.74 ± 0.86	3.81 ± 0.62
Marital status	Without partner	3.88 ± 0.63	3.05 ± 0.65	3.57 ± 0.87	3.70 ± 0.73	3.34 ± 0.65	2.48 ± 0.76	3.11 ± 0.96	3.28 ± 0.78
	With partner	3.95 ± 0.61	3.15 ± 0.61	3.84 ± 0.82	3.86 ± 0.69	3.46 ± 0.62	2.64 ± 0.73	3.52 ± 0.92	3.46 ± 0.75
Children	Yes	3.90 ± 0.63	3.14 ± 0.63	3.74 ± 0.82	3.83 ± 0.69	3.42 ± 0.64	2.65 ± 0.74	3.44 ± 0.92	3.43 ± 0.74
	No	3.94 ± 0.61	3.09 ± 0.62	3.74 ± 0.86	3.78 ± 0.72	3.42 ± 0.63	2.54 ± 0.74	3.33 ± 0.97	3.37 ± 0.77
Family monthly income	≤1 MW	3.71 ± 0.71	2.85 ± 0.60	3.30 ± 0.91	3.38 ± 0.74	3.27 ± 0.65	2.43 ± 0.75	3.00 ± 1.05	3.06 ± 0.77
	>1 to 2 MW	3.84 ± 0.63	2.97 ± 0.68	3.72 ± 0.97	3.45 ± 0.78	3.32 ± 0.62	2.40 ± 0.70	3.23 ± 1.08	2.93 ± 0.78
	>2 to 3 MW	3.81 ± 0.64	2.98 ± 0.66	3.64 ± 0.85	3.54 ± 0.76	3.31 ± 0.63	2.47 ± 0.77	3.21 ± 0.95	3.10 ± 0.82
	>3 to 5 MW	3.85 ± 0.61	3.04 ± 0.62	3.65 ± 0.88	3.67 ± 0.66	3.34 ± 0.65	2.51 ± 0.74	3.28 ± 0.94	3.24 ± 0.68
	>5 to 10 MW	3.96 ± 0.61	3.16 ± 0.62	3.80 ± 0.81	3.92 ± 0.66	3.45 ± 0.63	2.62 ± 0.73	3.46 ± 0.94	3.53 ± 0.71
	>10 to 20 MW	4.08 ± 0.59	3.30 ± 0.56	3.92 ± 0.76	4.14 ± 0.53	3.57 ± 0.57	2.75 ± 0.71	3.57 ± 0.88	3.76 ± 0.64
	>20 MW	4.06 ± 0.54	3.25 ± 0.56	3.76 ± 0.81	4.08 ± 0.62	3.62 ± 0.66	2.80 ± 0.72	3.50 ± 0.86	3.78 ± 0.69
Area of Practice	Clinic	3.95 ± 0.61	3.12 ± 0.61	3.75 ± 0.83	3.76 ± 0.71	3.45 ± 0.61	2.57 ± 0.70	3.36 ± 0.95	3.34 ± 0.74
	Teaching	4.05 ± 0.56	3.30 ± 0.56	4.00 ± 0.69	4.15 ± 0.52	3.44 ± 0.62	2.71 ± 0.75	3.61 ± 0.91	3.74 ± 0.62
	Foodservice administration	3.89 ± 0.61	3.08 ± 0.59	3.73 ± 0.84	3.74 ± 0.71	3.41 ± 0.64	2.56 ± 0.74	3.38 ± 0.92	3.36 ± 0.80
	Public health	3.90 ± 0.67	3.19 ± 0.62	3.80 ± 0.89	3.82 ± 0.70	3.32 ± 0.63	2.63 ± 0.69	3.32 ± 0.94	3.44 ± 0.64
	More than one area of practice	3.90 ± 0.63	3.07 ± 0.65	3.68 ± 0.85	3.78 ± 0.73	3.41 ± 0.66	2.56 ± 0.78	3.34 ± 0.94	3.37 ± 0.80
	Others	3.92 ± 0.62	3.10 ± 0.67	3.73 ± 0.95	3.80 ± 0.66	3.49 ± 0.51	2.65 ± 0.65	3.43 ± 1.02	3.36 ± 0.71
Number of workplaces	1	3.90 ± 0.63	3.09 ± 0.62	3.74 ± 0.85	3.77 ± 0.71	3.39 ± 0.64	2.57 ± 0.75	3.36 ± 0.97	3.37 ± 0.77
	2	3.96 ± 0.60	3.14 ± 0.63	3.75 ± 0.82	3.84 ± 0.69	3.42 ± 0.60	2.57 ± 0.72	3.39 ± 0.92	3.39 ± 0.74
	3	3.96 ± 0.57	3.20 ± 0.61	3.77 ± 0.84	3.92 ± 0.70	3.54 ± 0.59	2.64 ± 0.71	3.34 ± 0.92	3.59 ± 0.76
	>3	4.04 ± 0.72	3.14 ± 0.68	3.68 ± 0.89	3.87 ± 0.69	3.66 ± 0.72	2.71 ± 0.84	3.47 ± 0.93	3.56 ± 0.75
Type of institution	Private	3.92 ± 0.64	3.09 ± 0.66	3.73 ± 0.91	3.75 ± 0.71	3.43 ± 0.66	2.57 ± 0.77	3.37 ± 0.96	3.34 ± 0.77
	Public	3.93 ± 0.60	3.14 ± 0.59	3.76 ± 0.77	3.86 ± 0.70	3.41 ± 0.61	2.60 ± 0.71	3.37 ± 0.94	3.45 ± 0.75
Time undergraduate completion	≤2 years	3.93 ± 0.62	3.00 ± 0.64	3.67 ± 0.92	3.62 ± 0.72	3.38 ± 0.63	2.43 ± 0.76	3.21 ± 0.99	3.21 ± 0.77
	>2 to 5 years	3.95 ± 0.59	3.06 ± 0.63	3.73 ± 0.87	3.64 ± 0.78	3.44 ± 0.63	2.61 ± 0.74	3.42 ± 0.99	3.26 ± 0.79
	>5 to 10 years	3.95 ± 0.63	3.11 ± 0.64	3.80 ± 0.84	3.87 ± 0.72	3.42 ± 0.61	2.49 ± 0.71	3.39 ± 0.96	3.42 ± 0.78
	>10 to 15 years	3.89 ± 0.63	3.18 ± 0.67	3.73 ± 0.85	3.90 ± 0.66	3.39 ± 0.66	2.69 ± 0.78	3.43 ± 0.93	3.52 ± 0.75
	>15 years	3.91 ± 0.63	3.20 ± 0.55	3.77 ± 0.76	3.93 ± 0.61	3.45 ± 0.63	2.68 ± 0.70	3.43 ± 0.88	3.53 ± 0.69
Do you continue working during Sars-Cov-2?	no	3.89 ± 0.68	2.98 ± 0.66	3.68 ± 0.89	3.63 ± 0.73	3.31 ± 0.66	2.45 ± 0.74	3.29 ± 0.97	3.16 ± 0.80
	yes in person	3.86 ± 0.60	3.05 ± 0.60	3.68 ± 0.83	3.65 ± 0.66	3.37 ± 0.58	2.52 ± 0.71	3.32 ± 0.91	3.22 ± 0.69
	yes in person with some adaptations	3.98 ± 0.64	3.12 ± 0.66	3.73 ± 0.89	3.83 ± 0.73	3.50 ± 0.62	2.51 ± 0.76	3.36 ± 0.98	3.45 ± 0.78
	yes remotely	3.93 ± 0.60	3.20 ± 0.59	3.82 ± 0.79	3.94 ± 0.67	3.43 ± 0.64	2.66 ± 0.74	3.45 ± 0.94	3.56 ± 0.73
Did you test positive for Sars-Cov-2?	No	3.92 ± 0.62	3.12 ± 0.62	3.74 ± 0.85	3.81 ± 0.71	3.42 ± 0.63	2.59 ± 0.74	3.37 ± 0.95	3.40 ± 0.76
	Yes	3.89 ± 0.59	2.99 ± 0.69	3.78 ± 0.76	3.72 ± 0.61	3.33 ± 0.67	2.41 ± 0.77	3.50 ± 0.84	3.24 ± 0.72
Did any family members test positive for Sars-Cov-2?	No	3.92 ± 0.63	3.11 ± 0.63	3.72 ± 0.86	3.81 ± 0.72	3.43 ± 0.64	2.60 ± 0.75	3.36 ± 0.96	3.42 ± 0.77
	Yes (does not live with me)	3.86 ± 0.56	3.08 ± 0.69	3.85 ± 0.69	3.80 ± 0.70	3.27 ± 0.62	2.44 ± 0.71	3.39 ± 0.85	3.29 ± 0.78
	Yes (living with me)	3.96 ± 0.58	3.13 ± 0.60	3.80 ± 0.79	3.77 ± 0.61	3.40 ± 0.59	2.56 ± 0.70	3.44 ± 0.92	3.32 ± 0.70
**TOTAL**		3.93 ± 0.05 ^a^	3.12 ± 0.10 ^b^	3.75 ± 0.06 ^c^	3.81 ± 0.12 ^cd^	3.43 ± 0.05 ^a^	2.59 ± 0.15 ^b^	3.38 ± 0.12 ^a^	3.4 ± 0.11 ^a^

Different lowercase letters inside the last line comparing the four domains ^a,b,c,d^ (before and during the pandemic) show statistically different results (*p* < 0.05).

## Data Availability

Data sharing is not applicable to this article.
